# A Paper-Based Test for Screening Newborns for Sickle Cell Disease

**DOI:** 10.1038/srep45488

**Published:** 2017-04-03

**Authors:** Nathaniel Z. Piety, Alex George, Sonia Serrano, Maria R. Lanzi, Palka R. Patel, Maria P. Noli, Silvina Kahan, Damian Nirenberg, João F. Camanda, Gladstone Airewele, Sergey S. Shevkoplyas

**Affiliations:** 1 Department of Biomedical Engineering, University of Houston, Houston, Texas, USA; 2 Department of Pediatrics, Section of Hematology-Oncology, Baylor College of Medicine, Houston, Texas, USA; 3 Sickle Cell Newborn Screening Laboratory, Angola Sickle Cell Initiative, Cabinda City, Angola; 4 Global Health Corps, Baylor College of Medicine, Houston, Texas, USA; 5 Universidade Onze de Novembro Medical School, Cabinda City, Angola

## Abstract

The high cost, complexity and reliance on electricity, specialized equipment and supplies associated with conventional diagnostic methods limit the scope and sustainability of newborn screening for sickle cell disease (SCD) in sub-Saharan Africa and other resource-limited areas worldwide. Here we describe the development of a simple, low-cost, rapid, equipment- and electricity-free paper-based test capable of detecting sickle hemoglobin (HbS) in newborn blood samples with a limit of detection of 2% HbS. We validated this newborn paper-based test in a cohort of 159 newborns at an obstetric hospital in Cabinda, Angola. Newborn screening results using the paper-based test were compared to conventional isoelectric focusing (IEF). The test detected the presence of HbS with 81.8% sensitivity and 83.3% specificity, and identified SCD newborns with 100.0% sensitivity and 70.7% specificity. The use of the paper-based test in a two-stage newborn screening process could have excluded about 70% of all newborns from expensive confirmatory testing by IEF, without missing any of the SCD newborns in the studied cohort. This study demonstrates the potential utility of the newborn paper-based test for reducing the overall cost of screening newborns for SCD and thus increasing the practicality of universal newborn SCD screening programs in resource-limited settings.

Universal newborn screening, in combination with early intervention for affected infants, has nearly eliminated early childhood mortality due to sickle cell disease (SCD; HbSS) in high-income developed countries^1^,^2^,^3^. In contrast, a majority of children born with SCD in low-income developing countries die before the age of 5 due to lack of early diagnosis and comprehensive care^4^,^5^. Recent estimates suggest that implementation of wide-spread screening and follow-up care in countries affected most by the disease (e.g. Nigeria, Democratic Republic of the Congo, India) could save the lives of nearly 10 million children by 2050^6^. Data from pilot screening and treatment programs in sub-Saharan Africa demonstrate >95% survival for affected infants enrolled in inexpensive follow-up care, such as penicillin prophylaxis, pneumococcal immunizations, malaria bed nets, and family education about the disease^7^.

The major barriers limiting the expansion of these highly successful pilot screening programs are the high cost and technical complexity of conventional diagnostics methods (e.g. HPLC, high-performance liquid chromatography^8^, or IEF, isoelectric focusing electrophoresis^9^), and their dependence on specialized equipment, stable infrastructure and well-trained laboratory personnel^4^,^5^,^10^. Because of these limitations, newborn screening for SCD remains confined to only a few specialized clinical laboratories in major population centers, almost entirely missing the majority of all births occurring out of hospital^7^,^11^,^12^. Importantly, the results of screening with conventional laboratory methods are rarely available before postnatal discharge, reducing the all-important follow-up rate to as low as 50% of identified newborns^7^,^11^. There is therefore an urgent need for a screening test that is sufficiently inexpensive, portable, simple and rapid enough to enable universal screening of newborns for SCD in resource-limited settings lacking established infrastructure, specialized equipment or trained personnel.

We have previously described the development and clinical validation of a rapid, low-cost paper-based diagnostic test for SCD that permits the diagnosis of adults and children older than 6 months of age^13^,^14^,^15^. We now describe a test based on the same principles which is optimized to detect the low levels of sickle hemoglobin (HbS) present in the blood of newborns, allowing the direct screening of newborns for SCD and sickle cell trait (SCT; HbAS). We also report on the feasibility and diagnostic accuracy of the paper-based newborn test as performed by local health workers in a resource-limited clinical setting in Cabinda, Angola.

## Results

### Design and operation of the paper-based newborn screening test

**Figure 1:**
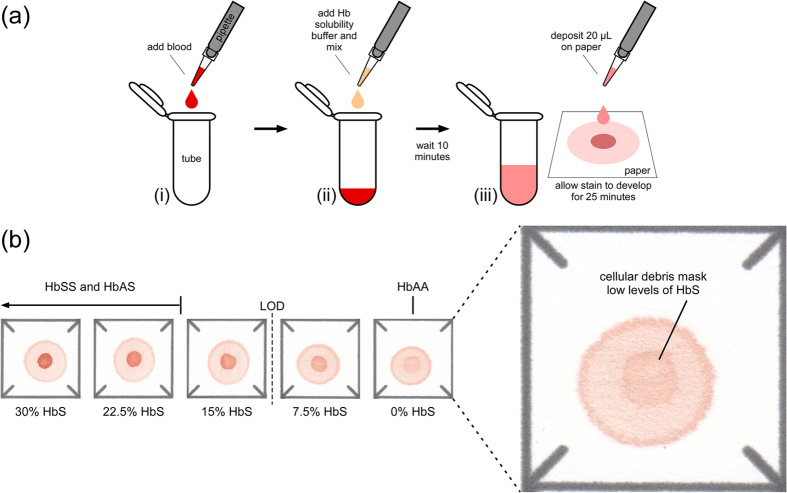
Previously developed paper-based SCD diagnostic test for adults and children older than 6 months of age. (**a**) Schematic illustration of the steps required to perform the adult paper-based test: (i) 20 μL of whole blood collected via finger-stick (or venipuncture) is added to the plastic tube; (ii) the blood is mixed with 200 μL of hemoglobin solubility buffer (phosphate buffer, saponin and sodium hydrosulfite); (iii) after 10 minutes, a 20 μL drop of the mixture is deposited on chromatography paper and allowed to dry for 25 minutes. (**b**) Representative bloodstains for samples with less than 30% sickle hemoglobin (HbS) produced by the adult paper-based test. Cellular debris remaining after RBC lysis create a faint center spot even for samples with 0% HbS, reducing the limit of detection (LOD; dashed line) for this version of the paper-based to ~15% HbS. Typical adult HbS ranges for different genotypes are marked above the stains.

The design and operation of a paper-based SCD test capable of diagnosing SCD in adults and children older than 6 months have been described previously in detail (Fig. 1a)^13^,^14^,^15^. When evaluated visually, the limit of detection (LOD) for the *adult* test was about 15% HbS^15^, which was insufficient for detecting the very low percentages of HbS typically found in newborn samples −6.5 ± 2.8% for SCT (HbFAS) and 10.2 ± 3.9% for SCD (HbFS)^16^. The reason for the relatively high LOD was the faint center spot in the center of the stain formed by cellular debris of lysed red blood cells even in the complete absence of any HbS (Fig. 1b, inset)^14^.

**Figure 2:**
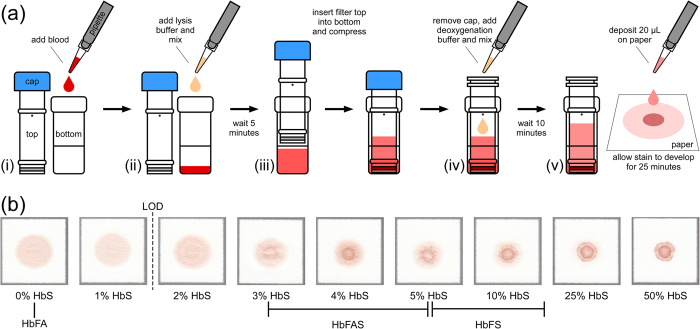
Paper-based newborn SCD screening test. (**a**) Schematic illustration of the steps required to perform the newborn paper-based test: (i) 20 μL of whole blood collected via heel-stick is added to the bottom chamber of the syringeless filter; (ii) the blood is mixed with lysis buffer (phosphate buffer and saponin); (iii) after 5 min the top of the syringeless filter is inserted into the bottom and compressed to filter out cellular debris; (iv) deoxygenation buffer (phosphate buffer and sodium hydrosulfite) is added to the filtered solution; (v) after 10 min a 20 μL drop of the mixture is deposited on chromatography paper and allowed to dry for 25 minutes. All steps required to perform the test can be completed within 40 min. Test results can also be read out visually, immediately after the formation of the blood stain. (**b**) Representative bloodstains produced on paper by the paper-based newborn SCD screening test for blood samples with various sickle hemoglobin (HbS) levels. The limit of detection (LOD; dashed line) for detecting the presence of any HbS in a blood sample visually was 2%. Typical newborn HbS ranges for different genotypes are marked below the stains.

To address this limitation and further lower the LOD of the test, we introduced a filtration step for removing cellular debris from the blood lysate prior to deoxygenation (Fig. 2a). This *newborn* paper-based test is performed by first mixing 40 μL of blood with 200 μL of lysis buffer (8 g/L saponin in phosphate buffer) in the bottom chamber of a disposable syringe-less filter (0.2 μm PES Whatman Mini-Uniprep™, GE Healthcare, USA). After 5 minutes, the filter top is compressed until the solution is filtered into the top chamber. An approximately equal volume (75 μL) of the deoxygenation buffer (200 g/L sodium hydrosulfite in phosphate buffer) is then added to the top chamber and mixed with the filtered solution. After 10 minutes, a 20 μL drop of the mixture is deposited onto chromatography paper (Whatman™ 1 Chr, GE Healthcare, USA) and allowed to dry for up to 25 minutes (Fig. 2a). The characteristic bloodstain pattern, indicative of the presence or absence of HbS in the blood sample, is formed by polymerized deoxy-HbS, which becomes trapped in the pores of the chromatography paper substrate in the area where the drop was deposited, and soluble forms of hemoglobin which wick laterally through the pores of the paper towards the periphery. The inherent red color of hemoglobin is sufficient for visual evaluation of the bloodstain pattern, with no additional signal amplification necessary.

Figure 2b shows characteristic bloodstain patterns for artificially reconstituted samples with 0, 1, 2, 3, 4, 5, 10, 25 and 50% HbS that were used to determine the LOD of the newborn test. Novice scorers (n = 5) with no previous experience performing or evaluating the test were provided with reference images of bloodstain patterns formed by samples with and without HbS and asked to score a set of stains (n = 45) as positive or negative for the presence of HbS. All users correctly scored all stains with >1% HbS as HbS-positive (LOD = 2% HbS). The test was independently performed 5 times for each sample in order to determine reliability of visual scoring. All users made the same diagnosis for all 5 tests at every HbS concentration, indicating that visual scoring of the test is highly reliable. The scoring process was also repeated 3 times for the same bloodstains (presentation order randomized) and scores were perfectly consistent between trials, further supporting that the visual scoring of the test is highly repeatable. The Fleiss’ kappa value over all scored stains was 1.0, suggesting a perfect agreement between all five scorers in interpreting the set of bloodstain patterns.

### Clinical validation of the newborn paper-based screening test in a resource-limited setting

**Figure 3:**
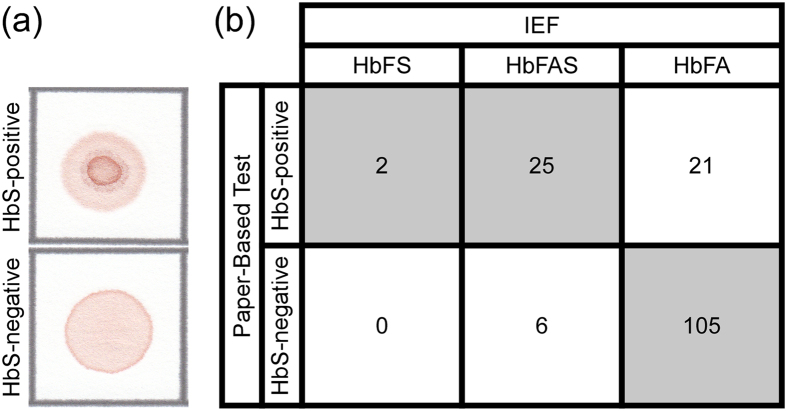
Accuracy of the paper-based newborn SCD screening test. (**a**) Representative bloodstains produced on paper by HbS-positive and HbS-negative newborn samples. (**b**) Confusion matrix of the results of tests performed and interpreted by local health workers at the newborn screening laboratory of the Clinica de Celulas Falciformes at the Dispensario Materno Infantil (Cabinda, Angola). Shaded cells contain numbers of correctly screened newborns (i.e. true HbS-positives and true HbS-negatives).

To evaluate the performance of the newborn paper-based screening test in the real-world environment of a resource-limited clinical setting, we deployed the test at the pilot newborn screening program in Cabinda, Angola. The rationale for this field testing was that newborn screening with the paper-based test should identify all samples positive for HbS (i.e. from both SCD and SCT newborns) and should therefore capture all newborns at risk for either disease or trait for confirmatory IEF testing. We trained local health workers to collect capillary blood samples from newborns via heel-stick, process the samples using the paper-based test, and score the result of the test visually as either HbS-positive or HbS-negative (Fig. 3a). The results of paper-based testing were then compared with those of IEF testing performed on the same samples as part of the current standard screening practice^7^.

Figure 3b shows the confusion matrix for the paper-based test performed by the local health workers on samples from 159 newborns with unknown SCD status. The test was able to identify HbS-positive samples with a sensitivity of 81.8% (95% confidence interval: 75.1–87.0%), specificity of 83.3% (CI: 76.8–88.3%), positive predictive value of 56.3% (CI: 48.5–63.7%), negative predictive value of 94.6% (CI: 89.9–97.2%) and overall diagnostic accuracy of 83.0% (CI: 76.4–88.1%). The relatively high number of false-positives for the newborn test – samples from normal (HbFA) newborns misidentified as HbS-positive – was due to occasional filter malfunction resulting in incomplete removal of cellular debris from the whole blood lysate, thus allowing the formation of a faint center spot which was misinterpreted as HbS-positive. All of the false HbS-negatives (4%) for the newborn test (i.e. samples containing some HbS misidentified as HbS-negative) were from SCT (HbFAS) newborns (Fig. 3b). Importantly, the newborn paper-based test was able to identify SCD (HbFS) newborns with a sensitivity of 100.0% (CI: 97.6–100.0%), specificity of 70.7% (CI: 63.2–77.2%), positive predictive value of 4.2% (CI: 2.0–8.5%), negative predictive value of 100.0% (CI: 97.6–100.0%) and overall diagnostic accuracy of 71.1% (CI: 63.6–77.6%).

**Figure 4:**
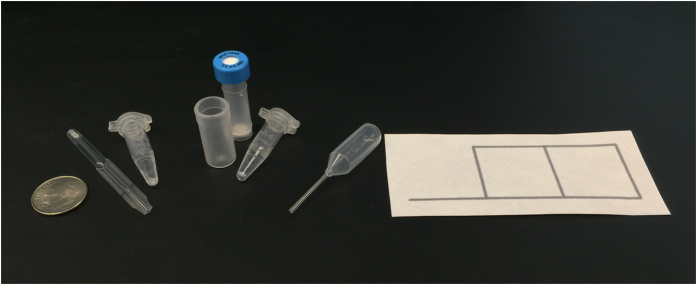
Self-contained, distributable version of the paper-based SCD newborn screening test kit, comprised of off-the-shelf components. The kit consists of one exact volume pipette for blood collection, two tubes containing lysis and deoxygenation buffers respectively, one syringeless filter, one dropper for depositing the mixture on paper, and one patterned piece of chromatography paper. Ten cent U.S. coin shown for size reference (17.9 mm diameter).

The paper-based test deployed in Angola employed a reusable pipette with disposable pipette tips (4 per test) for liquid metering. The cost per test for all test specific consumables (i.e., chromatography paper, reagents, syringeless filter, tubes and pipette tips) of the paper-based test deployed in Angola was $2.16 (for a detailed cost breakdown please see Supplementary Table 1). Figure 4 shows a self-contained, distributable version of the test kit comprised of off-the-shelf components, with about the same cost per test. For the self-contained test kit, the reusable pipette with disposable tips was replaced with disposable plastic pipettes and droppers. The total cost per test kit comprised of off-the-shelf materials is $2.12 (for a detailed cost breakdown please see Supplementary Table 2). The retail-priced syringeless filter and disposable plastic components constitute the majority of test cost (reagents and chromatography paper cost <$0.07 per test), and as such the per test cost can be expected to decrease significantly if the test kits are produced in large quantities using non-marked-up plastic components. The per test cost estimates for both versions of the paper-based newborn SCD screening test do not include the cost of consumables which would be common to any blood test (e.g. gloves, alcohol swab, lancet, collection tube and bandage), as these materials are not typically included in retail test kits.

Table 1 shows the potential cost savings from using the paper-based test as a preliminary screen-in test prior to confirmatory IEF testing of positive samples, based on the previously published cost per sample of $4.94 for IEF in Angola^7^. For our cohort of 159 newborns, screening all samples by IEF alone would cost $785.46 (cost per sample = $4.94). In contrast, screening using the paper-based test followed by IEF testing only of positive samples would cost an estimated $343.44 for the paper-based testing and $237.12 for subsequent IEF testing of positive samples, for a total cost of $580.56 (cost per sample = $3.65); a potential cost reduction of 29%. The relatively low incidence of SCD and SCT in the general population and consequently the relatively low ratio of confirmatory IEF to paper-based screening tests which will need to be performed, suggest that the potential cost savings from such a two-stage, screen-in program would increase proportionally with the number of newborns screened.

## Discussion

Two major barriers to successful implementation of universal newborn screening for SCD in low-income developing countries are the cost and logistical complexity of conventional diagnostic methods and the delayed availability of screening results^17^. In Angola, for example, the cost of newborn screening using IEF is reported as $4.94 per sample, and as a result screening remains limited to only the major urban centers of Luanda and Cabinda City^7^. The need to transport blood samples from birthing centers to a centralized testing laboratory for analysis also severely limits the ability to screen children at remote and rural facilities. In Angola, the results of IEF testing are rarely available less than 4 weeks after sample collection, and as a consequence, fewer than 55% of newborns with SCD identified through the IEF-based screening in Angola are successfully re-contacted and initiated on prophylaxis regimens^7^.

The current paradigm of universal newborn screening is to test *every* child for SCD at birth using a highly-accurate laboratory method such as IEF^18^. Given the relatively low incidence of SCD in the general population, most of the resources of such a screening paradigm are currently spent on diagnosing *healthy* children. A more cost-effective alternative could be to first identify newborns at the highest risk for having SCD using a rapid and low-cost screening test with a low false-negative rate, and then perform higher-cost confirmatory testing only for these screened-in high-risk newborns.

The paper-based SCD newborn screening test described here successfully addresses many of the technical and logistical impediments of the conventional approaches described above. Firstly, the per test cost of the self-contained, distributable paper-based test using off-the-shelf components purchased at retail prices is $2.12, already less than half the per test cost of conventional IEF currently employed in the pilot newborn screening program in Angola ($4.94 per sample). Additionally, the per test cost of the finalized test kit could potentially be lower than the current version as it will employ integrated sample collection and processing components, produced by a contract manufacturing partner, which are projected to be less expensive than the sum of the current retail prices for the multiple commercially available components comprising the current test. Secondly, the paper-based test is lightweight, completely electricity-free, requires no specialized equipment or instrumentation and is simple enough for a user with no previous experience performing the test to learn to operate and interpret the test with expert proficiency in under one hour. Therefore the paper-based test would not be limited to centralized laboratories with specialized equipment and trained technicians, but rather could be deployed to remote facilities and operated by health workers with any level of experience. In this study the results of the test were typically available within one hour or less of initiating the test. This rapid turnaround time, compared to conventional laboratory methods, could therefore potentially enable counseling of families with high-risk newborns before postpartum discharge (usually within 6–12 hours of delivery at the Primero de Maio obstetric center). Finally, our data show that a two-stage approach consisting of preliminary screening of all infants and confirmatory testing of only the high-risk subset could reduce the number of newborns requiring IEF testing by at least 70%, thus significantly reducing the overall cost of the screening program. More importantly, accurate identification of newborns at the highest risk for SCD could help focus limited available resources on establishing proper longitudinal care for this much smaller cohort.

The paper-based test offers important advantages over existing technologies designed to enable low-cost SCD diagnostics in resource-limited settings. Conventional hemoglobin solubility tests (e.g. SickleDex^TM^) are not sensitive enough to detect the low levels of HbS typically found in newborn blood samples, are notoriously difficult to standardize and interpret, and are confounded by numerous comorbidities which effect the turbidity of blood samples^19^. Various modifications of conventional laboratory methods (e.g., lower-cost implementation of IEF^20^, or a microfluidics-based HE^21^) as well as novel diagnostic approaches (e.g., density-based separation of sickle RBCs in capillaries^22^,^23^, or magnetic levitation-based smartphone platforms^24^) may reduce the cost of testing and/or may be performed at the point-of-care but continue to require highly trained personnel and rely on complex specialized equipment and electricity to operate. Rapid diagnostic tests for SCD based on conventional lateral flow immunoassay technology are instrument- and electricity-free and show great potential^25^,^26^,^27^. However, these tests still require extensive field-testing to determine real-world performance for newborn samples and for tests performed in resource-limited settings, and are subject to well-known limitations of all antibody-based assays, such as limited shelf-lives when ambient temperatures exceed recommended ranges for even short periods of time during shipping, storage or usage – a scenario which is highly likely in resource-limited setting such as sub-Saharan Africa^28^. Additionally, because these tests rely on proprietary antibodies, their cost per test may not be significantly lower than the cost of conventional testing methods such as IEF, and the rates and scale at which these tests can be manufactured may be limited by the rates at which the proprietary antibodies can be produced.

There are three major limitations to the paper-based newborn SCD screening test. The first is that the paper-based test cannot yet distinguish between SCT and SCD in newborns, and therefore must be used as a *screening*, rather than a *diagnostic* test. To make a definitive diagnosis, the screening results must be confirmed with a laboratory test (such as IEF) or later in life using the previously developed adult version of the paper-based test^13^,^15^. The second limitation is the relatively high incidence of false-positives observed in this study. This problem was due to the occasional malfunction of the filter used to remove cellular debris from the blood lysate, resulting in a false central spot mimicking that produced by HbS. The volume of blood and lysis buffer used in the filtration step has since been decreased in order to reduce the rate of false-positive results due to filter malfunction (please see Supplementary Information). Finally, we had a small but significant rate of false-negative results (4%) with the paper-based test, all of which were from newborns with SCT. A retrospective analysis of these samples and bloodstains revealed that the blood samples had clotted before processing or were otherwise inadequate, suggesting that these false-negatives were due to improper sample handling, rather than a malfunction of the paper-based test itself. Quality control standards for blood samples to be used with the paper-based test and obligate IEF analysis of samples deemed inadequate for the paper-based test could prevent these false negatives in further iterations of this two-stage testing protocol.

In summary, we demonstrate that the paper-based newborn SCD screening test, optimized to detect the low levels of sickle hemoglobin present in newborn blood, enables the direct screening of newborns for sickle cell trait and sickle cell disease. We also demonstrate that this test is feasible and has a high diagnostic accuracy when performed by local health workers in a resource-limited clinical setting. These results demonstrate the potential utility of the newborn paper-based test for identifying high-risk newborns in the immediate postnatal period and reducing the overall cost of screening newborns for sickle cell disease, thus increasing the practicality and effectiveness of universal newborn SCD screening programs in resource-limited settings.

## Methods

### Study design and participants

**Figure 5:**
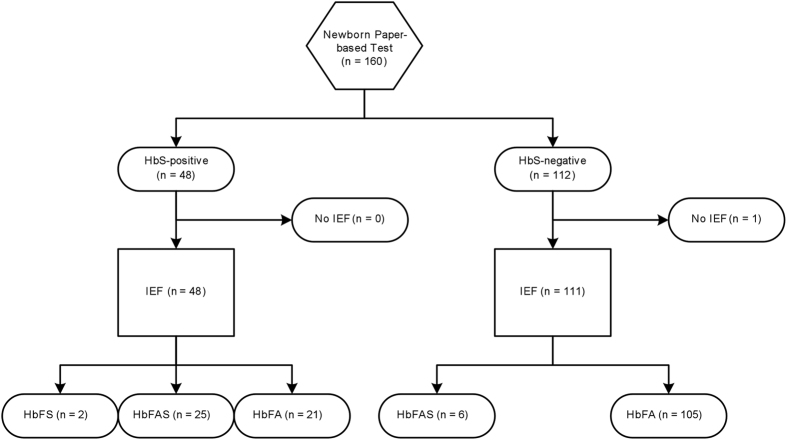
Classification flowchart for newborn samples evaluated with the paper-based SCD newborn screening test and isoelectric focusing (IEF).

All experimental protocols involving human blood samples were approved by the institutional review boards at the Universidade Onze de Novembro Medical School (Cabinda, Angola), Baylor College of Medicine (Houston, USA) and University of Houston (Houston, USA). Informed consent was obtained from all subjects. All experiments were performed in accordance with guidelines and regulations established by the University of Houston, Baylor College of Medicine and the U.S. Department of Health and Human Services for the protection of human study subjects. After obtaining informed consent from the newborns’ mothers, local health workers collected blood samples from newborns at the obstetric center of the Primero de Maio hospital in Cabinda. Eligibility criteria included gestation of greater than 30 weeks and uncomplicated delivery (judged by local clinical staff). Samples were collected from n = 160 newborns, a convenience sample. We successfully screened 160 newborn samples with the paper-based test, and performed successful IEF testing on 159 of these samples (Fig. 5).

### Blood sample collection, storage, and processing

For initial development of the paper-based newborn SCD screening test, blood samples were collected from SCD (HbSS) patients at the Texas Children’s Hematology Center and from healthy volunteers (HbAA) into Vacutainer vials (K_2_EDTA, BD Diagnostics, USA) using standard venipuncture technique. Samples were stored at 4 °C until use. Artificially reconstituted samples with a range of HbS levels were created by mixing ABO/Rh-matched, equal-hematocrit HbAA and HbSS blood samples at various ratios.

For test validation in Cabinda, blood samples were collected from newborns by heel-stick onto blood collection cards (Whatman 903 Protein Saver Card, GE Healthcare, USA) and into capillary blood collection tubes (Microvette, K_2_EDTA, Sarstedt AG & Co, Germany). Eluted dried blood spot samples were tested with isoelectric focusing (IEF) following existing standard operating procedures^7^. Liquid blood samples were refrigerated and used to perform the paper-based test within 7 days of collection. For all patients, the paper-based test was completed before IEF analysis. Local health workers interpreted the results of the paper-based test visually, using reference images of HbS-positive and HbS-negative bloodstains.

### Statistical analysis

Mean, standard deviation, p-values, and confusion matrices were calculated using built-in functions in MATLAB 2014b (The Math Works Inc., Natick, MA). 95% confidence intervals (CI) were calculated using the Wilson method^29^. Fleiss’ kappa calculations were performed to determine inter-operator agreement for visual scoring of bloodstains^30^. Performance metrics were calculated as: sensitivity = TP/(TP + FN); specificity = TN/(FP + TN); positive predictive value = TP/(TP + FP); negative predictive value = TN/(TN + FN); and accuracy = (TP + TN)/(TP + FP + TN + FN); where TP = true positive, FP = false positive, TN = true negative and FN = false negative.

## Additional Information

**How to cite this article:** Piety, N. Z. *et al*. A Paper-Based Test for Screening Newborns for Sickle Cell Disease. *Sci. Rep.* **7**, 45488; doi: 10.1038/srep45488 (2017).

**Publisher's note:** Springer Nature remains neutral with regard to jurisdictional claims in published maps and institutional affiliations.

## Supplementary Material

Supplementary Information

## Figures and Tables

**Table 1:** Potential cost savings from using the paper-based SCD newborn screening test as a preliminary screen, followed by confirmatory diagnosis of only screened-in HbS-positive newborns via isoelectric focusing (IEF).

	IEF alone	Paper-based test + IEF
Cohort size	159	159
Number screened by paper-based test	0	159
Total cost of paper-based screening (at $2.16/test)	$0	$343.44
Number requiring IEF testing	159	48
Total cost of IEF testing (at $4.94/test)	$785.46	$237.12
Total cost of all testing	$785.46	$580.56
Average cost per sample	$4.94	$3.65
