# Supplementary material for: A Paper-Based Test for Screening Newborns for Sickle Cell Disease
Source: Sci Rep. 2017 Apr 3;7:45488. doi: 10.1038/srep45488 (PMC5377336; doi:10.1038/srep45488)
Supplement: Supplementary Information [file srep45488-s1.pdf]

A Paper-Based Test for Screening Newborns for Sickle Cell Disease

Nathaniel Z. Piety,[†] Alex George,[†] Sonia Serrano, Maria R. Lanzi, Palka R. Patel, Maria P. Noli, Silvina Kahan, Damian Nirenberg, João F. Camanda, Gladstone Airewele, and Sergey S. Shevkoplyas*

[†] These authors contributed equally to this work.

* Corresponding author.

Supplementary Information

Supplementary Experiments:

The effect of lysate volume on rate of false positive results due to filter malfunction.

The volumes of blood, lysis buffer and deoxygenation buffer used to perform the paper-based test were decreased from 40 µL, 200 µL and 75 µL, respectively, for the original test to 15 µL, 75 µL and 40 µL, respectively, for the modified version of the test, in order to decrease the rate of false positive results due to filter malfunction. Importantly, the ratio of blood to lysis buffer (1:5 by volume) and the ratio of filtrate to deoxygenation buffer (1:1 by volume) were the same for both versions of the test. The total volume to be filtered in the modified version of the test was 40 µL which is only 10% of the listed filtering capacity of the syringeless filters (400 µL).

Artificially reconstituted samples (n = 5) with various HbS concentrations (0, 2, 4, 5% HbS) were used to determine the limit of detection of the modified version of the test. Blood samples with 0% HbS (n = 5) were used to determine the rate of false positives due to filter malfunction for the modified version of the test. The syringeless filters were compressed under various loading conditions (e.g. a finger, thumb, palm, fist, or Whatman 6 position multi-compressor, Thomson 8 position multi-use press), for a total of 79 repetitions under the different conditions.

We found that the limit of detection for the modified version of the test was the same as the limit of detection for the original test (LOD = 2% HbS). The rate of false positive results due to filter malfunction for the original test was 21 out of 126 (16.7%). The rate of false positive results due to filter malfunction for the modified version of the test was 5 out of 79 (6.3%). False positive results due to filter malfunction were only observed when the syringeless filters were compressed with palm; all other loading conditions produced no false positive results. By

decreasing the volumes of blood, lysis buffer and deoxygenation buffer used to perform the paper-based test, the rate of false positive results due to filter malfunction was decreased more than 2.5-fold overall, while the limit of detection of the modified version of the test remained that same as that of the original test.

Supplementary Table 1

Component	Purchase volume (units)	Cost per unit at purchase volume (USD)	Number of units used per test	Cost per test (USD)
Syringeless filter	100	$1.81	1	$1.81
Pipette tip	1000	$0.055	4	$0.22
Tube	1000	$0.03	2	$0.06
Chromatography paper	100	$0.50	0.1	$0.05
Subtotal	-	-	-	*$2.14*
**Component**	**Purchase volume (g)**	**Cost per gram at purchase volume (USD)**	**Number of grams used per test**	**Cost per test (USD)**
Potassium phosphate monobasic	1000	$0.125	0.063	$0.008
Potassium phosphate dibasic	10000	$0.099	0.083	$0.008
Saponin	25	$3.288	0.0016	$0.005
Sodium hydrosulfite	2000	$0.057	0.015	$0.001
Subtotal	-	-	-	*$0.02*
**Total**	**-**	**-**	**-**	**$2.16**

Supplementary Table 1 Detailed cost breakdown of consumable components and reagents comprising the paper-based test which employs a reusable pipette with disposable pipette tips for liquid metering.

Supplementary Table 2

Component	Purchase volume (units)	Cost per unit at purchase volume (USD)	Number of units used per test	Cost per test (USD)
Syringeless filter	100	$1.81	1	$1.81
Dropper	1000	$0.10	1	$0.10
Exact volume pipette	1000	$0.08	1	$0.08
Tube	1000	$0.03	2	$0.06
Chromatography paper	100	$0.50	0.1	$0.05
Subtotal	-	-	-	*$2.10*
**Component**	**Purchase volume (g)**	**Cost per gram at purchase volume (USD)**	**Number of grams used per test**	**Cost per test (USD)**
Potassium phosphate monobasic	1000	$0.125	0.063	$0.008
Potassium phosphate dibasic	10000	$0.099	0.083	$0.008
Saponin	25	$3.288	0.0016	$0.005
Sodium hydrosulfite	2000	$0.057	0.015	$0.001
Subtotal	-	-	-	*$0.02*
**Total**	**-**	**-**	**-**	**$2.12**

Supplementary Table 2 Detailed cost breakdown of consumable components and reagents comprising the paper-based test which employs disposable pipettes and droppers for liquid metering.
